# The Emerging Epigenetic Role of CD8+T Cells in Autoimmune Diseases: A Systematic Review

**DOI:** 10.3389/fimmu.2019.00856

**Published:** 2019-04-18

**Authors:** Qiancheng Deng, Yangyang Luo, Christopher Chang, Haijing Wu, Yan Ding, Rong Xiao

**Affiliations:** ^1^Hunan Key Laboratory of Medical Epigenetics, Department of Dermatology, The Second Xiangya Hospital, Central South University, Changsha, China; ^2^Department of Dermatology, Hunan Children's Hospital, Changsha, China; ^3^Division of Rheumatology, Allergy and Clinical Immunology, University of California, Davis, Davis, CA, United States; ^4^Department of Dermatology, Hainan Provincial Dermatology Disease Hospital, Haikou, China

**Keywords:** epigenetics, CD8+T cells, DNA methylation, histone modification, MiRNAs, autoimmune diseases

## Abstract

Autoimmune diseases are usually complex and multifactorial, characterized by aberrant production of autoreactive immune cells and/or autoantibodies against healthy cells and tissues. However, the pathogenesis of autoimmune diseases has not been clearly elucidated. The activation, differentiation, and development of CD8+ T cells can be affected by numerous inflammatory cytokines, transcription factors, and chemokines. In recent years, epigenetic modifications have been shown to play an important role in the fate of CD8+ T cells. The discovery of these modifications that contribute to the activation or suppression of CD8+ cells has been concurrent with the increasing evidence that CD8+ T cells play a role in autoimmunity. These relationships have been studied in various autoimmune diseases, including multiple sclerosis (MS), systemic sclerosis (SSc), type 1 diabetes (T1D), Grave's disease (GD), systemic lupus erythematosus (SLE), aplastic anemia (AA), and vitiligo. In each of these diseases, genes that play a role in the proliferation or activation of CD8+ T cells have been found to be affected by epigenetic modifications. Various cytokines, transcription factors, and other regulatory molecules have been found to be differentially methylated in CD8+ T cells in autoimmune diseases. These genes are involved in T cell regulation, including interferons, interleukin (IL),tumor necrosis factor (TNF), as well as linker for activation of T cells (LAT), cytotoxic T-lymphocyte–associated antigen 4 (CTLA4), and adapter proteins. MiRNAs also play a role in the pathogenesis of these diseases and several known miRNAs that are involved in these diseases have also been shown to play a role in CD8+ regulation.

## Introduction

Autoimmune diseases are complex diseases characterized by the loss of immunological tolerance to self-antigens and sustained aberrant immunological response against healthy cells and tissues, leading ultimately to the overproduction of autoreactive immune cells and/or autoantibodies (1, 2). However, the pathogenesis of autoimmunity is still not well understood (3). It is becoming evident that both the innate and the adaptive immune response are involved in the pathogenesis of autoimmune diseases (4), especially adaptive immune response. Adaptive immune cells, including B and T cells, have been demonstrated to be primary contributors to the overactive immune response and overproduction of antibodies. Traditionally, the roles of B lymphocytes and CD4+ T lymphocytes in autoimmune diseases have already been widely studied and are well recognized. However, mounting evidence has suggested that CD8+ T cells, in particular, play an important role in the induction, progression, pathogenesis, and protection for autoimmune diseases (5).

CD8+ T cells, also called cytotoxic T lymphocytes (CTL), are one subtype of T cell (6), characterized by robust production of interferon (IFN)-γ and cytolytic activities via perforin (PRF)/granzymes (GZM) or Fas mechanisms to kill target cells (7). In autoimmune diseases, these target cells killed by autoreactive CD8+ T cells can release numerous autoantigens to induce the overproduction of autoantibodies, and finally lead to the death of self-cells. This produces a local inflammatory response as well. The activation, differentiation, and development of CD8+ T cells is accompanied by large-scale changes in the coordinated expression of numerous inflammatory cytokines, transcription factors (TFs) and chemokines that are correlated with their survival, effector function, and self-renewal (7). However, in recent years, the role for epigenetics in CD8+ T cells has been increasingly recognized, with a bulk of the currently available evidence demonstrating the significance of epigenetic modifications in the fate of CD8+ T cells (8).

Epigenetics refers to stable and heritable changes in gene expression without involving changes in DNA sequence, which are thought to be connection between environment factors and genetics (9). Epigenetic mechanisms play a crucial role in controlling gene expression during cell growth, development and differentiation, as well as in response to environmental factors (10). Nevertheless, epigenetic changes can lead to a gene dysregulation which can result in various pathological conditions such as autoimmune diseases and/or cancers (11). Three primary mechanisms of epigenetic modifications include DNA methylation, histone modifications and micro-RNAs (miRNAs) (12).

In summary, this review aims to highlight the epigenetic role of CD8+ T cells in the pathogenesis of autoimmune diseases.

## CD8+ T Cells

### Generation of CD8+ T Cells

**Figure 1:**
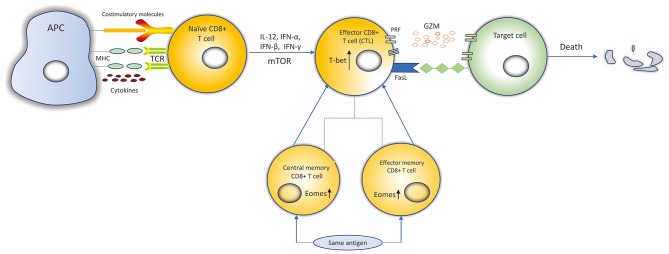
Differentiation and killing mechanisms of CD8+ T cells. Naïve CD8+ T cells expressing the TCRs recognize MHC-I: peptide presented by APCs. Naïve CD8+ T cells are activated. IL-12, IFN-α, IFN-β, IFN-γ, and mTOR can promote the expansion, survival and development of naïve CD8+ T cells to differentiate into CTLs. Transcription factors, such as Eomes, T-bet, increase proliferation of memory CD8+ T cells and cytolytic function of CTLs. CTLs kill target cells by secreting cytolytic granules (PRF and GZM) or recognizing the Fas receptors expressed on the target cell surface to induce the death of target cell. Most of the CTLs experience apoptosis and the remaining CTLs differentiate into central memory and effector memory CD8+ T cells. When re-encountering the same antigens, they can be activated and differentiate into CTLs to kill target cells.

According to their activation status, CD8+ T cells can be divided into four subtypes: naïve, effector, central memory and effector memory CD8+ T cells (13, 14). When encountering antigens, naïve CD8+ T cells express the cell surface T-cell receptors (TCRs) that can recognize class I major histocompatibility complex (MHC class I) presented by antigen-presenting cells (APCs) (15). Then, naïve CD8+ T cells differentiate into effector CD8+ T cells and go through expansion. Data suggests that naïve CD8+ T cells probably undergo up to 19 cell divisions in a week after pathogen stimulation, generating a potential 500,000-fold expansion (14, 16). Effector cells possess the ability of cytotoxicity to kill targeted cells. 90–95% of effector CD8+ T cells experience apoptosis when targeted cells have been cleared. However, the remaining effector CD8+ T cells then differentiate into central memory T (T_CM_) and effector memory T (T_EM_) cells, and return into a resting state (17). When re-encountering the same antigens, the T_EM_ T cells can be promptly activated, and then differentiate into effector CD8+ T cells to mediate a new immune response (Figure 1). T_CM_ cells are more gradually activated under the same circumstances (13, 17).

### Functions of CD8+ T Cells

Previous studies have been demonstrated the development and character of CD8+ T cells were mainly generated by the research of viral infections, such as hepatitis B virus (HBV). HBV-specific CD8+ T cells are the principal contributor factor in the viral elimination and liver inflammation. HBV-specific CD8+ T cells are significantly increased in HBV-infected liver to kill infected hepatocytes by secreting cytotoxic granules (PRF/GZM) and IFN-γ or by Fas/FasL and recruiting other immune cells. CD161 and CXCR6 receptor play an essential role in the migration of CD8+ T cells into liver parenchyma (18). It is important to note that in addition to releasing cytotoxic granules (PRF/GRMB) and cytokines such as IFN-γ to kill target cells at the sites of infection, effector CD8+ T cells play a regulatory role in producing immunosuppressive cytokine interleukin (IL)-10 to limit tissue damage (14, 19). In addition, CD8+ T cells are the most important cells to clear tumor cells through specifically recognizing tumor antigens. It is emerging that activated CD8+ T cells release extracellular vesicles (20) including various cytokines, mRNAs, microRNAs, chemokines and TFs to interrupt tumor invasion and metastasis (21). Recently, it has been reported that GZMB released by CTLs has an additional function to aid CTLs transmigrating into tumor lesions by mediating basement membrane remodeling (22). In addition to the protective function of CD8+ T cells in viral infections and tumors, evidence primarily from studies in the experimental autoimmune encephalomyelitis (EAE), multiple sclerosis (MS), systemic lupus erythematosus (SLE), type 1 diabetes (T1D), rheumatoid arthritis (RA), and vitiigo have been manifested CD8+ T cells play an essential part in the progression of cell and tissue-specific autoimmune disease (13).

CD8+ regulatory T (23) cells are a highly immune suppressive subtype of CD8+ T cells, characterized by suppressing the activation and proliferation of reactive effector cells via cell-to-cell contact (24). Unlike CD4+ Treg cells, studies on CD8+ Treg cells are relatively less (25). Recently, it has been found that CD8+ Treg cells play a regulatory role in autoimmune disease (26, 27), infectious diseases (28, 29) and tumors (25, 30) as well. Several cell markers have been observed on the CD8+ Treg cells, such as forkhead box P3 (Foxp3)+, CD25+, CD122+, CD103+, and CD28-(25). The exhausted markers such as cytotoxic T-lymphocyte–associated antigen 4 (CTLA4), lymphocyteactivation gene 3 (LAG3), PD-1, and glucocorticoid-induced tumor necrosis factor receptor -related (GITR) are also expressed on CD8+ Treg cells. Among these markers, Foxp3 is a specific marker and the master regulatory molecule of Tregs (24, 31). Foxp3+CD8+ Treg cells are dependent on the expression of CTLA4 to suppress effector T-cell responses *in vitro* (27). It has been observed that soluble factors, such as IL-10 and/or transforming growth factor beta (TGF-β), or cell–cell contact are mainly involved in the suppressive activity of Treg cells (25). However, further studies are needed to explore the mechanisms that are implicated in the induction of CD8+ Treg cells.

### The Influence of Cytokines, Chemokines, and TFs on CD8+ T Cells

The fate of CTLs can be influenced by numerous inflammatory cytokines, TFs, and chemokines. Many inflammatory cytokines such as IL-12, IFN-α, and IFN-β, are able to promote the expansion, survival and development of cytotoxicity. IFN-γ can also promote expansion (15, 32). T-bet is a T-box TF, encoded by *Tbx*21, that contributes to the expression of IFN-γ, PRF and GZMB in cytotoxic CD8+ T cells (33). Eomesodermin (Eomes), also a type of T-box TF, is highly expressed in memory cells. Studies suggested that T-bet and Eomes may provide cooperative functions in the genes encoding IFN-γ, PRF and GZM in CD8+ T cells (34). Eomes^+/−^*Tbx*21^−/−^ mice are defective in IFN-γ induction and impaired in cytolytic function in CD8+ T cells (33). Chemokine receptors are essential for CD8+ T cells to egress from the blood, then enter specific target issues, and are capable of identifying CTLs with distinct functions (35). CX3CR1 expressed on effector CD8+ T cells may correlate with their potential to generate memory subtypes (36). Studies have suggested that CXCR3 and its ligand CXCL10 are useful for CD8+ T cells to discover their targets in lymph nodes and encephalitic tissue, respectively (35, 37). Evidence suggests that CCR7 is necessary for the recruitment of CTLs to viral infection sites in the brain (38). CXCL16 constitutively expressed in keratinocytes was found to mediate the homing of CD8+ T cells in human skin. Li et al. demonstrated that the CXCL16-CXCR6 chemokine pair mediates CD8+ T cells migration under oxidative stress in patients with vitiligo, and blocking the CXCL16-CXCR6 interaction undermines CD8+ T cells' trafficking (39).

## Epigenetics

### DNA Methylation

**Figure 2:**
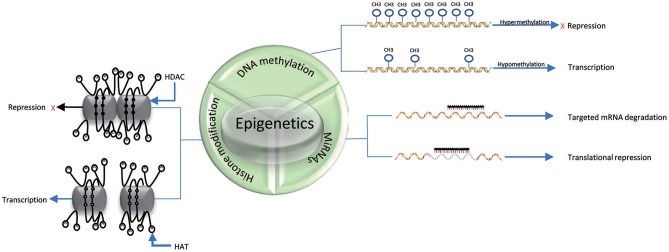
Mechanisms of epigenetics. DNA hypermethylation leads to the repression of gene expression, while DNA hypomethylation promotes gene transcription. Histone deacetylation (D) of histone tails catalyzed by HDACs in association with DNA methylation (black solid circle) represses gene expression; Acetylation of histone tails (A) regulated by HATs in association with DNA demethylation (black hallow circle) promotes gene expression. miRNAs can suppress translation by binding to specific mRNAs. The three epigenetic modifications can interplay with each other.

**Figure 3:**
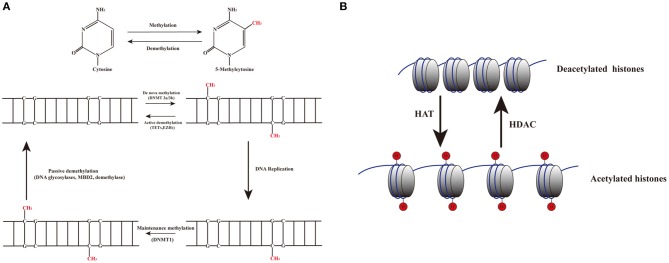
Dynamic mechanisms of DNA methylation and demethylation. **(A)** The addition of a methyl group to the 5th carbon in cytosine residues of cytosine-guanine (CpG) dinucleotides produces 5-methylcytosine residues. DNMT3a and DNMT3b are involved in *de novo* methylation; DNMT1 maintains epigenetic covalent modifications during DNA replication. DNA demethylation can be aroused actively or passively. Passive demethylation is induced by the failure of maintenance methylation after DNA replication. Active methylation is caused by replication-independent processes. **(B)** Histone acetylation is dynamically catalyzed by HATs by transferring acetyl groups to lysine, which leads to an open conformation of chromatin permitting gene expression. Deacetylation is implicated in repressing gene expression by HDACs via removing the acetyl groups.

DNA methylation is an epigenetic process that involves the addition of a methyl group to the 5th carbon in cytosine residues of cytosine-guanine (CpG) dinucleotides, to produce 5-methylcytosine residues (10). This reaction is mediated by DNA methyltransferase enzymes (DNMT) including DNMT1, DNMT3a, DNMT3b, and DNMT3L (9). 5-methylcytosine is synthesized by DNMTs through transferring of a methyl group donor from S-adenosylmethionine (SAM) to cytosine (40). DNMT1 is responsible for maintaining epigenetic covalent modifications during DNA replication and repair, while DNMT3a and DNMT3b are involved in *de novo* methylation during embryonic development. DNMT3L acts on embryogenesis (41). It is generally accepted that DNA methylation results in silencing of gene expression through two fundamental mechanisms. One is that methylation of cytosine bases directly decreases the affinity for binding of TFs. An additional mechanism involves methylated DNA-binding domain (MBD) that are recruited to methylated CpG sequences to alter chromatin structure to form a co-repressor complex, thereby leading to the repression of gene transcription. DNA demethylation promotes gene transcription (42, 43) (Figure 2). DNA demethylation can be aroused actively or passively. Passive demethylation is induced by inhibition of DNMTs that can occur during DNA replication (9, 44, 45) DNA can be actively demethylated by a broad range of molecules, such as DNA glycosylases, MBD2, demethylase and glucocorticoid (44, 46). However, the molecular mechanisms are not clear. Active DNA demethylation implicates in oxidation of the methylated base via ten-eleven translocations (TETs), or the methylated deamination or a nearby base by activation induced deaminase (47). In addition, methyltrasferase EZH2 plays a novel role in the active demethylation by the combination of TET2 to form the DNA demethylation complex and the catalytically inactive DNMT3L (48) (Figure 3). Importantly, the interact between methylation and demethylation can maintain a specific cellular epigenetic state (49).

### Histone Modifications

Histones are conserved nuclear proteins that form the core center of the nucleosome. The nucleosome, which is the basic subunit of eukaryotic chromatin, is comprised of 146 base pairs (bp) of DNA wrapped around an octamer of two pairs of four core histones (H2A, H2B, H3, and H4) (50). Histone modifications include acetylation, methylation, ubiquitination, phosphorylation, sumoylation, citrullination, ADP-ribosylation, and proline isomerization (51). These modifications serve as signals, referred to as the “histone code,” which regulate the transcription process (2, 52). The most common histone modification is the acetylation and deacetylation of lysine residues (43). Histone acetylation is dynamically catalyzed by histone acetyltransferases (HATs) by transferring acetyl groups to lysine, which leads to an open conformation of chromatin permitting gene expression. In contrast, histone deacetylation is implicated in repressing gene expression by histone deacetylases (HDACs) via removing the acetyl groups (53) (Figures 2, 3). Histone methylation, another important mechanism of histone modification, is dependent on two enzymes, methylases and demethylases, regulating different biochemical reactions (40).

### MiRNAs

MiRNAs are a class of genome-encoded and non-coding RNAs consisting of 21–23 bps that function as post-translational regulators of gene expression (12). MiRNAs lead to translational suppression or degradation by binding to the 3′ untranslated region (UTR) of specific messenger RNAs (mRNAs) (11) (Figure 2). It is generally accepted that miRNAs have been found to regulate approximately 90% of protein-coding genes and play a critical role in biological processes, such as embryogenesis, cell differentiation, cell cycle progression, apoptosis, and immune functions (54). Growing evidence suggests that miRNAs have a close relationship and interplay with other mechanisms of epigenetics, such as DNA methylation and histone modifications (55). In fact, miRNAs themselves can regulate DNA methylation and histone methylation by interacting with gene transcripts, and the resulting changes in DNA methylation and histone modification can subsequent lead to epigenetic effects. Conversely, miRNA expression can be affected by DNA methylation and histone modifications. For example, DNMTs and HDACs inhibitors have been proposed to be able to upregulate miRNAs (56–58).

### Metabolism and Epigenetics

Epigenetic modifications play a crucial role in cellular state for the reason that it integrates the information induced by stimuli. Accumulating evidence manifests metabolic dynamics altered by stimuli exerts profound control of epigenetic mechanisms (59, 60). S-adenosylmethionine (SAM), acetyl-coenzyme A (acetyl-CoA), and nicotinamide adenine dinucleotide (NAD+) function as important metabolites, which can cooperate with epigenetic modifying enzymes, such as DNMTs, HDACs, and HATs, to alter the chromatin structure that ultimately promote or inhibit gene expression in autoimmune responses (61–63).

SAM is generated from adenosine triphosphate (ATP) and methionine by methionine adenosyltransferase (MAT) and a product of the one-carbon metabolism cycle (64, 65). DNA and histone methyltransferases enzymes are dependent on SAM as a methyl donor. Alterations in methionine metabolism can change SAM level and then directly affect trimethylation level of H3K4 and ultimately modulate gene expression (66). Reduced SAM level could negatively influence the activity of the histone demethylase (62). SAM and MTA can synergistically inhibit transmethylation level in lymphocytes and alleviate the T-cell-mediated manifestations in lupus (67). S-adenosylhomocysteine (SAH) is an important byproduct of SAM during methyltransferase reactions, which inhibits DNMTs and histone methyltransferases (HMTs) (68). The SAM/SAH ratio could act as a biosensor of the cellular metabolic state deciding the catalytic activity of methyltransferases enzymes (62, 69). Methionine metabolism level is a useful way to evaluate histone methylation level by regulating SAM and SAH (64). Changes in SAM and SAH levels have an effect to regulating the activity of the chromatin state (66). Additionally, evidence has shown that ratio of SAM/SAH can also influence the metabolic regulation of methionine and DNA methylation level (66). Study in rat liver has suggested that increased adenosine and homocysteine levels result in dramatic decrease in the ratio of SAM/SAH, then leading to the inhibition of SAM-dependent methyltransferase enzymes and a global reduction in DNA methylation (69–71).

Acetyl-CoA is generated primarily from glucose and fatty acid metabolism in the TCA cycle and is an important metabolite that contributes to cellular energy production, lipid metabolism (63, 72). Importantly, acetyl-CoA can be utilized by HATs to donate an acetyl group for lysine acetylation reactions and serves as substrates for the acetylation of histones (62, 63, 73). Glucose serves as a major source of acetyl-CoA. Therefore, high glucose in media increased intracellular acetyl-coA and histone acetylationv level, subsequently promoting gene expression (74, 75). Generally, high acetyl-CoA level is positively associated with global histone acetylation (76, 77). Acetyl-CoA functions as a biosensor of the celluar metabolic state influencing specific genes in response to nutrient availability via epigenetic modificaitons such as histone acetylation (62).

NAD+ is a substrate of the glycolytic pathway and a crucial cofactor to mediate the deacetylation reactions catalyzed by the highly conserved sirtuin family of HDACs (63, 78). NADH is a reducing agent of NAD+ (62). Sirtuin levels depend on the balance of NAD+ /NADH (79, 80). The NAD+ dependent sirtuins provide an important connection between metabolism and the epigenetic dynamics (81). For instance, circadian vibrations of NAD+ activate periodic fluctuations of SIRT1-mediated deacetylation of H3K9 and H3K14 at the promoters of genes (82). Moreover, a dramatic reduction of NAD+ level has negative effect on SIRT1 activity. Fluctuation of NAD+level, as a function of nutrient availability, has an impact on sirtuin catalysis largely supported by the research on SIRT1 (62). High-fat diet treated mice was found due to deficient SIRT1 activity resulting from NAD+ depletion (83–85). Histone acetylation level can be influenced by both calorie restriction and high-fat diet via NAD+ levels. Intriguingly, calorie restriction may potentially be promoted high NAD+/NADH and thus lead to deacetylation (86). In addition, sirtuin activity may potentially be inhibited by increased NADH due to hypoxia (87).

Though growing evidence of researches support the notion that metabolism positively or negatively influence chromosome structure (81). Epigenetic modifying enzymes maybe viewed as potential biomarkers to diagnose various diseases and also as new therapeutic targets for reversing the abnormal immune responses in autoimmune diseases. However, further studies are needed to explore how the metabolic pathways correlating with epigenetics contribute to the immune response.

## Epigenetic Regulations on CD8+ T Cells

Given the evidence that epigenetic mechanisms participate in the activation, differentiation, and development of CD8+ T cells, it is not surprising that the role for epigenetics in determining the fate of CD8+ T cells has been increasingly appreciated (40). There is an abundance of evidence supporting the epigenetic influence on the development and activity of naïve, effector and memory CD8+ T cells (8).

### DNA Methylation and CD8+ T Cells

DNA methylation plays an essential role in the maintaining the gene expression profile of mature T cells, as well as regulating the differentiation of T cells (88). DNMT1 is required for the normal clonal expansion, survival and function of CD8+ T cells during viral infections (89). Conditional knockout of DNMT1 during activation of CD8 T cells leads to a decreased effector pool, fewer memory cells and weaker cytolytic activity (90). Evidence suggests that DNMT1-mediated methylation at Foxp3 is required for its stable expression repression in CD8+ T cells (91). Epigenetic mechanisms can target cytokine genes in T cells. In naïve CD8+ T cells, IFN-γ is methylated at several of the CpG sites in the promoter, which results in the repression of gene expression (92). Furthermore, demethylation of the IFN promoter is associated with the expression of its mRNA and protein both in mice and humans (44). The expression of other effector genes, such as PRF and GZMB, have been shown to be significantly upregulated as a result of hypomethylation in the promoter regions of these genes in effector CD8+ T cells (90). Demethylation of TFs, such as c-JUN and NFATc1 contributes to the generation of effector CD8+ T cells (93). It is widely accepted that the mechanistic target of rapamycin (mTOR) functions as a serine/threonine-protein kinase regulating the metabolism, expansion, differentiation, trafficking, and survival of immunocytes, including CD8+ T cells (Figure 1), in response to different environmental stimuli (94–96). Díaz-Molina's team has found that IFN-γ PRF and FASLG (Fas ligand) were demethylated after early everolimus (a mTOR inhibitor) conversion in heart transplantation (HT) patients, which were closely associated with the increased CD8+ T_EM_ cells. These changes are beneficial to improve the anti- cytomegalovirus infection (CMV) immune response in HT patients (94).

### Histone Modification and CD8+ T Cells

The permissive or repressive histone modifications can alter the expression of effector and memory CD8+ T cells (89). Histone hyperacetylation has been observed at the promoters of IFN-γ, GZMB and PRF1, and their regulators in CD8+ memory T cells (97). Evidence indicates that induced hyperacetylation or hypoacetylation of these genes leads to an increasing or decreasing expression, respectively, in CD8+ T cells (97). Histone modification plays an important role in the expression of Eomes, PRF1, and GZMB in CD8+ T cells. Araki et al. found that increased expression of Eomes, PRF and GZMB is linked to enrichment of the proximal promoter and exon 1 on histone 3 in memory CD8+ T cells (98). Acetylated lysine 9 in histone H3 (H3K9Ac), enriched in active regulatory regions, such as promoter or enhancer regions in CD8+ T cell subsets, often experiences dynamic modifications associated with gene-expression (99). Maltby et al. demonstrated that after HDAC inhibitor (Trichostatin A) treatment, the expression of H3K9Ac and genes of naïve CD8 T cells increased, and that HDAC inhibitors may play an important role in regulating gene profile expression and functions of memory CD8+ T cells (100). For example, decreased H3K9Ac level reduces Eomes expression in memory CD8 T+ cells (98). Moreover, ACY241, another HDAC inhibitor, activates the AKT/mTOR/p65 pathways and upregulates transcription regulators including Bcl-6, Eomes, HIF-1 and T-bet, which result in increased proliferation and cytolytic function of antigen-specific CD8+ T_EM_ cells with stronger anti-tumor activities in multiple myeloma and solid tumors (101).

### MiRNAs and CD8+ T Cells

MiRNAs have also been shown to play a role in the fate of CD8+ T cells. MiR-181a has been reported to dampen the TCR signal threshold in CD4+CD8+ T cells with endogenous TCRs against self-antigens. Deficiency of miR-181a leads to high reactivity of peripheral T cells against self-antigens during immunization in a mouse model, suggesting that miR-181a-deficiency manifests T cells bearing TCRs with high self-reactivity (102). CD8+ T cells from miR34^−/−^ mice express decreased levels of CD69 and CD25 and less proliferation with reduced ERK phosphorylation than wild-type cells. Further reports demonstrated that stimulation of Jurkat T cells transduced with miR-34a increased CD69 expression and ERK signaling (103). In addition, miR-34a-deficient lymph node (LN) cell infusion impaired CD8+ cell expansion in a murine model for LN cell-induced bone marrow failure (104).

MiR-150 is a cell-intrinsic factor required for robust effector CD8+ T cell expansion and differentiation. It has been shown that naïve miR-150^−/−^ CD8+ T cells are unable to experience robust expansion, resulting in downregulated effector cells with decreased cytotoxicity (105). Another miRNA, miR-29 is highly expressed in adult naïve CD8+ cells and the downregulation of miR-29 upon activation leads to efficient cytolytic activities (106). Moreover, Yang et al. showed that miR-15a/16 deficiency mice resist the exhaustion and enhanced CD8+ T cell-mediated immune response to relieve glioma progression by influencing mTOR (107). The mTOR signal pathway also plays an important role on proliferation and functions of CD8+ T cells in allogeneic stem cell transplantation (SCT) patients. MiR-625-3p might serve as an essential role in the downstream cascade of TCR signals to promote CD8+ T cell proliferation. Expression of miR-625-3p can be inhibited by mTOR inhibitor rapamycin, then influencing CD8+ T cell proliferation and functions that mediating the anti-viral immunity and graft-vs.-host-disease (GVHD) in SCT patients. However, it is unclear whether miR-625-3p in CD8+ T cells was directly regulated by mTOR signal pathway (108). Interestingly, miR-140 functions as a tumor-suppressive role in osteosarcoma tumor, which might be correlated with increased CTLs and reduced myeloid-derived suppressive cells and Treg cells through inhibiting mTOR signal pathway with significant synergistic anti-tumor effect (109).

## The Epigenetic Regulation of CD8+ T Cells in Autoimmune Diseases

**Figure 4:**
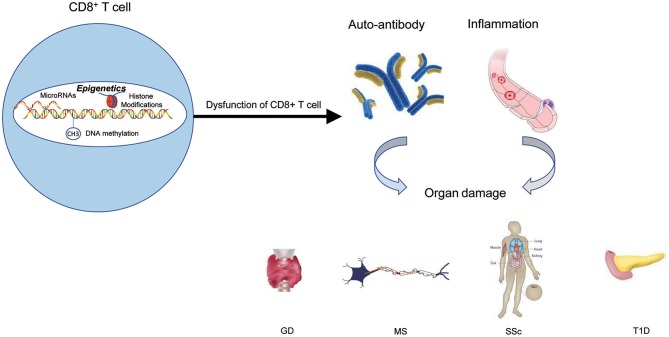
The epigenetic role of CD8+ T cells in autoimmune diseases. Epigenetic mechanisms participate in CD8+ T cells' activation, differentiation, and development, and finally lead to the dysfunction of CD8+ T cells. The results of dysfunction of CD8+ T cells can initiate abnormal CD8+ T-cell responses, thus triggering the production of autoantibodies and inflammation that lead to autoimmune diseases.

Over the years, the epigenetic role of CD4+ T lymphocytes in autoimmune has been increasingly well established. However, the role of CD8+ T cells is still not completely understood from a mechanistic standpoint. It is extremely likely that one of the ways to develop autoimmune diseases is through epigenetic modifications of CD8+ T cells. The contribution of this pathway to autoimmune diseases may exceed the role of genetic factors in the development of autoimmune diseases (Figure 4 and Table 1).

**Table 1:** The potential role of CD8+ T cells in different autoimmune diseases.

**Diseases**	**Level of CD8+ T cells**	**Potential role of CD8+ T cells**	**References**
GD	Decreased	Causes the production of intrathyroidal autoantibodies	(110, 111)
MS	Increased	Mediates inflammation	(112)
SSc	Increased	Contributes to the skin fibrosis	(113, 114)
T1D	Increased	Induces β-cell death	(115)
SLE	Increased	Induces autoantibody appearance and causing organ damage	(116, 117)
SAA	Increased	Causes hematopoietic cell health	(118)
Vitiligo	Increased	Mediates the destruction of melanocytes	(119)

### Grave's Disease (GD)

GD is an autoimmune thyroid disorder, characterized by the production of circulating autoantibodies that can stimulate the thyroid hormone receptor (TSH-R) on thyroid follicular cells, ultimately leading to goiter and hyperthyroidism (120, 121). Evidence has shown that thyroid autoreactive CD8+ T cells are important factors in the pathogenesis of GD (122).

CD8+ T cells were significantly downregulated in peripheral blood mononuclear cells(PBMC)of GD patients. CD8+ cells may suppress the activation B cells via a CD40 pathway to decrease CD23+ cells and production of IL-10. Moreover, CD8+ T cells may have an effect on the correction of the Th1/Th2 balance by reducing Th2 dominance (110, 111). However, it should be verified in other study of *vivo* and *vitro* in future. Limbach et al. have analyzed the role of CD8+ T cells by using a genome-wide analysis of DNA methylation in the GD and found 3322 differentially methylated CpG sites in CD8+ T cells. Hypermethylated CpG sites were enriched in genes involved in suppressing activation of CD8+ T cells, including Src family tyrosine kinase LCK, T cell receptor zeta chain CD247, its interacting Src kinase ZAP70, TCR associated CD3 family members (CD3D, CD3E, and CD3G), co-signaling receptors CTLA4 and PDCD1, TCR adapter proteins LAX1 and SLA2, and CD8A (123). The nuclear matrix binding transcriptional silencing factor SATB1, an epigenetic regulator of the T cell lineage and required for T cell's differentiation and survival, was the most significant transcriptional regulator of differentially methylated genes in CD8+ T cells. Moreover, genes encoding the C2H2-type zinc finger BCL11B, HLA class I component B2M, chemokine receptor CXCR4, adapter protein FYB, cytokine IFN-γ and tumor necrosis factor (TNF) receptor superfamily member TNFRSF1B, all with known functional roles in T cells, have methylation differentiation in CD8+ T cells of GD (123) (Table 2). Taken together, these genes may provide knowledge that can be applied for future therapeutic directions in GD.

**Table 2:** Epigenetic changes of CD8+ T cells in different autoimmune diseases.

**Diseases**	**Involved organs/cells**	**Epigenetic changes**	**Species**	**Functions**	**References**
GD	Thyroid	Hypermethylation of LCK, CD247, ZAP70, CD3D, CD3E, CD3G, CTLA4 and PDCD1, LAX1 and SLA2, and CD8A	Human	Disturbing the activation and regulation of CD8+ T cells	(123)
GD	Thyroid	Reduced H3K4me3 and H3K27ac of CD247, CD3E, CD3G, LCK, ZAP70, and CTLA4	Human	Involved in the activation of CD8+ T cells	(123)
GD	Thyroid	Reduced miRNA-200a_1 and miRNA-200a2*	Human	Assisting CD8+ T cells producing thyroid cell-specific antibodies	(122)
MS	CNS	Hypermethylation of MORN1		Not mentioned	(100)
MS	CNS	Increased miR-16, miR-155 and miR-142-3p	Human	Inhibiting Treg-suppressive activity	(124–126)
SSc	Skin	Hypomethylation of IFI44L, IFITM1, MX1, PARP9	Human	Activation of Wnt signaling	(127, 128)
T1D	β cells and pancreas	Increased miR-29b	Mice	Impairing the cytotoxicity of CD8+ T cells	(129)
SLE	Multi-organs	Hypomethylation of PRF	Human	Enhancing the cytotoxicity of CD8+ T cells	(56, 116)
SAA	Hematopoietic cells	Hypomethylation of LAT	Mice	Enhancing the cytotoxicity of CD8+ T cells	(130)
SAA	Hematopoietic cells	Increased histone H3 acetylation	Human	Enhancing the cytotoxicity of CD8+ T cells	(131)

Gene ontology (GO) analysis suggests that there are reduced H3K4me3 and H3K27ac signals at the relevant genes participated in T cell activation. In GD, Limbach et al. performed genome-wide profiling of H3K4me3 and H3K27ac in CD8+ T cells to identify differentially enriched regions. In line with their findings on DNA methylation profiling, they observed that genes such as CD247, CD3E, CD3G, LCK, ZAP70, and CTLA4 have decreased levels of H3K4me3 and H3K27ac in their promoters regions in the CD8 + T cells of GD patients, which potentially may impede the function of T cell receptor signaling to inhibit CD8 + T cells (123) (Table 2). However, the specific signal pathways need to be further studied.

Evidence shows that miRNA-200a_1 and miRNA-200a2^*^ in CD8+ T cells are significantly reduced in GD (122). The decrease of miRNA200a in CD8+ T cells may play a role in the development of thyroid cell-specific antibodies, including anti-thyroperoxidase (TPO) and anti-thyroglobulin (Tg), ultimately leading to the destruction of thyroid tissue by CD8+ T cells (122, 132) (Table 2).

### Multiple Sclerosis (MS)

MS is a chronic, degenerative autoimmune disease of the central nervous system (CNS), accompanied by axonal loss and inflammation contributing to a progressive neurodegenerative process (2).

CD8+ T cells were the most common T cells found in acute and chronic MS lesions, outnumbering CD4+ T cells by 3–10 fold in chronically inflamed MS plaques (6). Intriguingly, previous studies have shown that CD8+ T cells not only contribute to the pathogenesis of acute MS plaques, but are also implicated in the oligoclonal expansions in CNS, blood, and even cerebrospinal fluid (CSF) of MS patients (133–135). However, growing evidence shows that CD8+ T cells play a significant role in the downregulation of MS and experimental autoimmune encephalomyelitis (EAE) (112). Neuroantigen-specific CD8+ T cells are activated by APCs cross-presenting neuroantigens. The activated regulatory CD8+ T cells secrete large amounts of IFN-γ and PRF, which can result in the repression and/or cytotoxic killing of encephalitogenic CD4+ T cells that mediate inflammation (112).

Evidence suggests that CD8+ T cells have distinct DNA methylation profiles in MS (100). DNA methylation plays a critical role in multiple sclerosis by regulating levels of its target genes (136). In MS patients, 95% differentially methylated CpG-sites in CD8+ T cells were hypermethylation when compared to controls, such as *APC2, HOXA2, HRNBP3, HEXDC* and *NTRK3*. However, the role of DNA hypermethylation of CpG-sites within this gene in immune cells from MS patients is unclear. Since the role of DNA methylation at gene promoters is gene silencing, it is possible that these hypermethylated genes maybe inhibit the functions of CD8+ T cells to participate in the pathogenesis of MS (137). Moreover, further studies demonstrated that CD8+ T cells had a single hypermethylated CpG in the MORN repeat-containing protein 1 (MORN1) gene but at different sites within the gene in MS patients (100) (Table 2).

It has been reported that the expression of miR-16, miR-155 and miR-142-3p was upregulated in CD8+ T cells from MS patients by targeting FOXP3, interferon regulatory factor 2 binding protein 2 (IRF2BP2) and FOXO1, respectively (124). The overexpression of these miRNAs are involved in activating CD8+ T cells to mediate autoimmune inflammation (138, 139). In addition, Quach et al. showed that miR-181a was increased in tolerant CD8+ T cells in MS. However, this miRNA was only observed effects of CRYAB, a small heat shock protein, on cytokine secretion but not growth of CD8+ T cells (140). MiR-497, miR-30a-3p and miR-149 were decreased in CD8+ T cells in MS compared with normal controls. Target prediction based on the T-cell activation has not been experimentally validated (141). Other differentially expressed miRNAs such as miR-17-5p, miR-193, and miR-126 have been identified in CD8+ T cells from patients with MS (142) (Table 2). Nevertheless, the involvement of these dysregulated miRNAs on the CD8+ T cells in MS has largely remained unknown (143).

### Systemic Sclerosis (SSc)

SSc is a heterogeneous and life-threatening autoimmune and connective tissue disease characterized by over-production of autoantibodies, dysregulation of the immune system, endothelial cell dysfunction, deposition of extracellular matrix, activation of fibroblasts (FBs), and ultimately, fibrosis in the skin, tissues and/or organs (144, 145).

It has been demonstrated that CD8+ T cells are upregulated in the peripheral blood of SSc patients (113). Moreover, peripheral blood CD8+ T cells from SSc patients secrete high levels of IL-13, which is associated with upregulation of the transcription factor GATA-3 and maintenance of proper expression of IFN-γ, and is thus ultimately implicated in skin fibrosis (113, 114).

An abnormal type I IFN-associated pathway plays a critical role in the fibrosis, disease severity and progression of SSc. However, epigenetic alterations of type I IFN-associated pathways have not previously been observed in SSc FBs or immune cells. Global hypomethylation of type I IFN signaling pathway-associated genes, including interferon induced protein 44 like (IFI44L), interferon induced transmembrane protein 1 (IFITM1), MX dynamin like GTPase 1 (MX1), and poly (ADP-ribose) polymerase 9 (PARP9), has been found in CD8+ T cells of SSc patients, which may contribute to the pathogenesis of SSc (127) (Table 2). Among these genes, IFI44L is an IFN-inducible protein with uncertain function (146). Hypomethylation of this gene was identified as a potential biomarker for diagnosis in SLE (147). IFITM1 is implicated in host-pathogen interaction, and IFN-α and IFN-γ can induce expression of IFITM1 in response to pathogens (148). IFITM1 was demonstrated to be associated with Wnt signaling (128). Furthermore, activation of Wnt signaling has been shown to be involved in fibrosis in animal models and SSc patients (149). Therefore, epigenetic modification of IFI44L and IFITM1 in CD8+ T cells are worthy of further study in SSc. Moreover, researchers have observed that there is a significant association between gene expression and the methylation status of these IFN-associated genes in CD8+ T cells in SSc (127).

### Type 1 Diabetes (T1D)

T1D is a T lymphocyte mediated autoimmune disease, which ultimately results in the destruction of the β cells and pancreas (40). The majority of the infiltrating cells in the pancreas of patients with T1D have been shown to be CD8+ T cells and hyperexpression of MHC I molecules has been observed on β cells, which strongly supports the role of CD8+ T cells in T1D (150, 151). Interaction between CD8+ T cells and β cells can induce β-cell death and the amplification of autoreactive T cells in pancreatic islets (115).

In a murine model of adoptive transfer of autoimmune diabetes, downregulated miR-29b decreased the cytolytic activity of transferred CD8+ T cells (129, 152) (Table 2). In CD8+ T cells, miRNAs (miR-23b, miR-98, and miR-590-5p) can reduce the expression of several key apoptotic molecules, such as FAS, FASLG, TNF-related apoptosis-inducing ligand (TRAIL) and TRAIL-R2, indicating they can serve as modifiers of disease susceptibility in T1D patients (153). It has been shown that the repression of pro-apoptotic pathways (the Fas/FasL and TRAIL/TRAIL-R pathways) by microRNAs leads to unrestricted expansion of diabetogenic CD8+ T cells, suggesting microRNAs may mediate gene silencing in islet cell autoimmunity in T1D patients (153).

### Systemic Lupus Erythematosus (SLE)

SLE is an autoimmune disease characterized by multi-system involvement and overproduction of a variety of antinuclear autoantibodies (12, 154). It has been appreciated for some time that the pathogenesis of SLE is multi-factorial. Richardson' team has found some methylation-sensitive genes by DNA methylation inhibitor 5-azacytidine (5-azaC) treating PHA-stimulated T cells. MRNA expression was then compared in treated and control cells via oligonucleotide arrays. PRF was identified as a transcript in the hypomethylated cells. PRF was overexpressed in both CD4+ and CD8+ T cells due to hypomethylation of a conserved region located between the promoter and upstream enhancer (116, 155). The expression of PRF was abnormally increased in CD4+ T cells from patients with active lupus (116). In addition, increased numbers of CD8+ T cells expressing PRF and/or GZMB were observed in SLE, which may be caused by IFN α-activated dendritic cells (DCs) strongly altering the differentiation of CD8 T lymphocytes. Then CD8+ T cells can induce the production of nontolerized autoantigens. Importantly, this increase was related to disease severity (156). It has been reported that the increased PRF may be responsible for the autologous monocyte/macrophage killing in SLE patients (116). Subsequent increase in monocyte/macrophage apoptosis induces autoantibody development and causes organ damage (117). Taken together, the expression of PRF in CD8+ cells correlates closely with lupus via the methylation status of its promoter region (157). Conversely, SLE-associated DNA hypomethylation contributes to the overexpression of CD8+ T cells-specific PRF1 (56, 116) (Table 2).

Activation of mTOR has been demonstrated to contribute to the dysfunction of T cells in SLE patients (158). Sirolimus (a mTOR inhibitor) effectively reversed the exhaustion CD8+ memory T cells and expanded CD8 effector-memory T cells in patients with SLE (158). The mTOR complex 1 (mTORC1) activity increased significantly in CD4^−^CD8^−^ double-negative (DN) T cells, while mTORC2 activity diminished, primarily in CD8+ T cells (159). Diminished mTORC2 activity in CD8+ T cells may lead to the abnormal expression of FoxP3 in Treg cells (159, 160), which may result in the immunoregulation dysfuction to contribute to the pathology of SLE. CD4^+^CD25^+^FOXP3^+^ T-cells increased by blocking mTOR activity, probably via inhibiting T-cell activation-induced methylation (161). However, future studies are warranted to investigate the pathogenic and protective role of CD8+ T cells and how the regulation of CD8+ T cell by mTOR may be exerted through epigenetic mechanisms in SLE and other autoimmune diseases.

**Figure 5:**
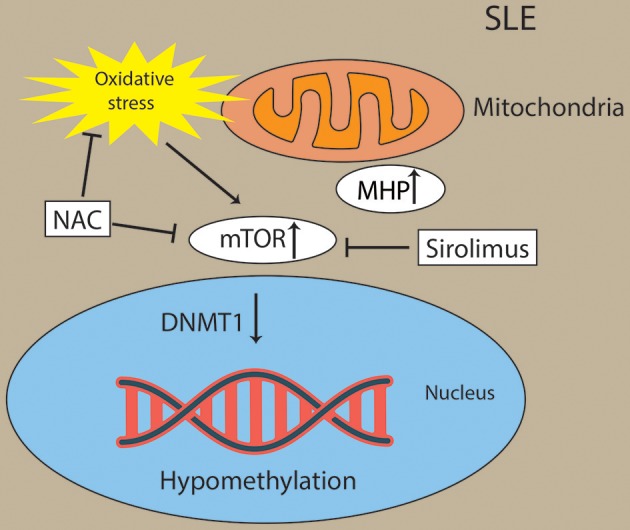
Activation of mTOR and oxidative stress drive Pathogenesis in lupus T cells. Oxidative stress originates from mitochondrial hyperpolarization (MHP) and activated the mTOR pathway in lupus T cells. Oxidative stress and mTOR and contribute to the hypomethylation in lupus T cells through diminishing activity of DNMT1. Sirolimus (a mTOR inhibitor) and NAC (an oxidative stress inhibitor) has been found to block the activity of mTORC1 in lupus T cells.

Oxidative stress activated the mTOR pathway in lupus T cells. Oxidative stress originates from mitochondrial hyperpolarization (MHP), which also contributed to abnormal T-cell activation in SLE patients (162–164). mTORC1 has been recognized as a oxidative stress sensor in SLE. N-acetylcysteine (NAC), an oxidative stress inhibitor, has been found to block the activity of mTORC1 in lupus T cells (162). Overproduction of reactive oxygen species (ROS) has been a primary contributor of hypomethylation in lupus T cells by preventing protein kinase Cδ and further diminishing the expression of DNMT1 (165, 166). For example, oxidative stress induced the expression of serine/threonine-protein phosphatase 2A catalytic subunit α (PP2ACA) isoform through reducing activity of DNMT1 and ultimately leading to demethylation of the promoter of PPP2CA to contribute to the pathology of SLE (167, 168) (Figure 5). However, future studies are needed to decipher how the interaction of oxidative stress and mTOR in CD8+ T cells contribute to the pathology of SLE via epigenetic mechanisms.

### Severe Aplastic Anemia (SAA)

SAA is an autoimmune disease with destruction of hematopoietic cells by activated T lymphocytes, especially CD8+ T cells (169). Linker for activation of T cells (LAT) is a transmembrane adaptor protein, which plays a significant role in the function of T cells. In SAA patients, the overexpression of LAT, which may result from antigenic stimulus or genetic mutation, may in turn strengthen CTL cytotoxicity and result in hematopoietic failure (118). The promoter of LAT has been shown to be hypomethylated in SAA as well (130). Thus, there may be a role for hypomethylation of LAT in enhancing CD8+ T cells' cytotoxicity in SAA. Studies have also shown that the differentiation and function of CD8+ T cells can be regulated by acetylation modification of histone H3. Qi et al. explored the CD8+ T cells' histone H3 acetylation level in the bone marrow of SAA patients and observed that histone H3 acetylation level in CD8+ T cells was dramatically and negatively correlated with the immune status and hematopoietic function in SAA patients (131) (Table 2). The hyperfunction of CD8+ T cells were associated with the high level of histone H3 acetylation, which resulted in the hematopoietic dysfunction of bone marrow and the exacerbation of disease condition in SAA patients (131).

## Concluding Remarks

Emerging studies provide sufficient evidence to demonstrate that CD8+ T cells play an important role in autoimmune diseases. There are increasing number of studies supporting the role of epigenetic modifications in the activation, differentiation, and development of CD8+ T cells. However, further studies are needed to better understand the epigenetic regulations of CD8+ T cells in the pathogenesis of autoimmune diseases. In addition, CD8 T cells and mTOR are new concepts and targets for SLE (170) and other autoimmune diseases. More studies are needed to focus on the relationship of CD8 T cell and mTOR and how the regulation of CD8 T cell by mTOR may be exerted through epigenetic mechanisms in autoimmune diseases. New technologies such as single-cell sequencing, single molecule real time sequencing (SMRT) and CRISPR/Cas9 can be used to study the role of epigenetics in CD8+ T cells in the pathogenesis of autoimmune diseases, and may result in the discovery of novel biomarkers for diagnosis, disease monitoring, and therapeutic validation.

## Author Contributions

QD wrote the manuscript. YL oversaw the editing. CC, HW, YD, and RX revised the manuscript. All authors read and approved the final manuscript.

### Conflict of Interest Statement

The authors declare that the research was conducted in the absence of any commercial or financial relationships that could be construed as a potential conflict of interest.
